# Correctness, Harmfulness, and Diversity of Large Language Models for Colonoscopy Preparation Assistance: Comparative Evaluation Study

**DOI:** 10.2196/88581

**Published:** 2026-08-04

**Authors:** Tomiris Kaumenova, Subhankar Chakraborty, Eric Fosler-Lussier, Kebire Gofar, Isaiah Metcalf, Andrew Perrault, Michael White

**Affiliations:** 1 Department of Linguistics, The Ohio State University, 1712 Neil Ave, Columbus, OH, 43210, United States; 2 Division of Gastroenterology, Hepatology and Nutrition, Department of Internal Medicine, The Ohio State University Wexner Medical Center, Columbus, OH, United States; 3 Department of Computer Science & Engineering, The Ohio State University, Columbus, OH, United States; 4 Department of Internal Medicine, Brookdale University Hospital Medical Center, Brooklyn, NY, United States; 5 College of Medicine, The Ohio State University, Columbus, OH, United States; 6 Novant Health New Hanover Regional Medical Center, Wilmington, NC, United States

**Keywords:** colorectal cancer, colonoscopy, health communication, virtual assistants, large language models

## Abstract

**Background:**

Colorectal cancer is a leading cause of cancer-related deaths in the United States, and colonoscopy remains the gold standard for early detection and prevention. However, many procedures are postponed due to inadequate bowel preparation, a preventable failure often caused by patients’ difficulty in understanding and following written prep instructions. Prior interventions such as reminder apps and instructional videos have improved adherence only modestly, largely because they cannot answer patient-specific questions. Recent advances in large language models (LLMs) raise the possibility of developing conversational assistants that can provide interactive support to patients in procedure preparation.

**Objective:**

This study evaluated the correctness, harmfulness, and diversity of synthetic dialogues generated by leading LLMs acting as both simulated AI Coaches and patients for colonoscopy preparation.

**Methods:**

Five leading LLMs—OpenAI’s o3, GPT-4.1, and GPT-5.1; Meta’s Llama 3.3 70B; and Mistral’s Large-2411—were used to generate 250 patient-AI Coach dialogues per model. Dialogues consisted of 3 to 7 question-answer pairs concerning diet, medications, and other prep-related topics. A multiprompt, multiquestion approach was designed to elicit diverse patient questions, and an error taxonomy was established to assess model capabilities in responding to questions. Human raters, including 3 medical experts, evaluated the generated questions for difficulty and the responses for correctness, error type, and potential harmfulness. Automatic evaluation using an LLM-as-a-judge approach complemented human evaluation. Question diversity was assessed using lexical diversity metrics (Distinct-1 and Distinct-2) and entropy. In addition, we evaluated a safety filtering mechanism in which responses judged incorrect by an automated evaluator were replaced with a deferral message instructing patients to contact their health care provider. Differences in response correctness across models were evaluated using permutation tests conducted at the dialogue level. Interrater agreement among human evaluators was assessed using the Gwet AC1 statistic. The study was conducted between May and September 2025.

**Results:**

Automatic evaluation results closely aligned with human judgments: leading models approached but did not achieve adequate performance. Closed-weight models (GPT-5.1, GPT-4.1, and o3) outperformed open-weight models (Llama and Mistral) on correctness, with the reasoning models (GPT-5.1 and o3) performing best. This turn-level ranking was preserved under the supplementary single-prompt baseline, although dialogue-level rankings differed. All models produced harmful errors, primarily due to omissions or misinterpretations of prep instructions. The multiprompt generation strategy substantially increased the diversity of patient questions compared with a single-prompt baseline. Applying an automated safety filter reduced overall error rates but failed to eliminate harmful responses.

**Conclusions:**

Although LLMs demonstrate strong potential to support colonoscopy preparation, none are yet reliable enough for unsupervised deployment in patient-facing contexts. Persistent harmful errors and the limited effectiveness of simple filtering mechanisms highlight the need for improved instruction adherence, stronger safety mechanisms, and validation using real patient queries.

## Introduction

Colorectal cancer is the fourth leading cause of cancer-related deaths in the United States [1], and colonoscopy remains the gold standard for early detection and prevention. Tens of thousands of colonoscopy and endoscopy procedures are performed each year at Ohio State’s Wexner Medical Center. However, despite their efficacy, around 20% of these tests are postponed because patients have not read, understood, and correctly carried out prep instructions. Inadequate colonoscopy prep has economic, health-related, and social costs. Economic costs include missed days from taking the prep, taking time off for the procedure, travel costs to get to the appointment and back, and the cost of having an accompanying person to drive (which is a requirement for the procedure). For the health care system, they lead to a missed appointment, incurring resultant wasted resources. Health-related costs include potentially reduced compliance with screening guidelines (only about 70% comply [2]) and thus risk of missing polyps. Social costs include frustration on the part of patients, in addition to patients sharing their experience with others, which can deter others from undergoing colonoscopy.

A key driver of this preventable problem is information overload: patients receive lengthy and complex written instructions days or weeks before their procedure, making it difficult to recall and correctly execute each step at the right time. For example, patients are given the information sheets that typically instruct them to drink half of a prep solution for clearing out the colon at 6 PM on the evening before their scheduled procedure and the other half 6 hours before the procedure time. However, some patients nevertheless show up for their procedure with the second half of their prep solution in hand—falsely assuming that they are supposed to take it after arriving at the procedure facility—and end up having to reschedule the procedure.

Past interventions, such as reminder apps [3], instructional videos, and automated text messages [4], have improved prep adherence only modestly [5], largely because they lack the capacity for interactive question answering. The ability to answer questions appears to be critical: when automated systems could not respond to patient questions [6], improvements in adherence disappeared [7]. Thus, a conversational assistant that can safely respond to patient queries is a promising next step in supporting patients during colonoscopy preparation.

Recent progress in large language models (LLMs) makes this prospect newly feasible. Frontier models such as o3 and Med-PaLM 2 [8] have demonstrated strong reasoning on clinical benchmarks, including OpenAI’s HealthBench [9] and MedQA [10]. If these models can perform complex diagnostic reasoning, a natural question arises: how well can they handle the simpler task of colonoscopy preparation coaching? This task primarily tests prompt adherence, prep instruction understanding, temporal and common sense reasoning, and dialogue communication, rather than clinical inference. Our study attempts to address this question. Building on prior work by Arya et al [11], which introduced a neuro-symbolic conversational guide for colonoscopy prep, we explore how LLMs perform on the same challenge. Our preliminary experiments show that recent LLMs substantially outperform earlier models, particularly on temporal reasoning and conversational abilities, which are core difficulties in the task. This motivates a systematic evaluation of LLMs.

A growing body of work has emerged that explores capabilities and limitations of LLMs in medical question-answering tasks and patient-facing contexts. In nutrition, Sun et al [12] have evaluated ChatGPT as an AI dietitian for type 2 diabetes. In mental health, LLMs were assessed on postpartum depression frequently asked questions (FAQs), with responses evaluated using the GRADE (Grading of Recommendations Assessment, Development, and Evaluation) framework [13]. In oncology, LLMs were tested on both FAQs [14] and patient queries in an electronic patient portal [15]. One study found that health care professionals evaluated chatbot responses to patient questions from an online forum as empathetic and high quality [16], and other work has shown that patients were more satisfied with LLM-generated responses than with clinicians’ responses to their questions asked through electronic health records (EHR) [17]. Another comprehensive study spanning 17 specialties [18] evaluated LLM responses to physician-generated questions for correctness and completeness. Relatedly, the CRAFT-MD (Conversational Reasoning Assessment Framework for Testing in Medicine) framework [19] demonstrated how simulated patient-provider interactions can be used to systematically evaluate LLMs across a wide range of clinical tasks. These studies highlight the need for evaluating LLMs in specialized patient-facing and question-answering contexts, such as colonoscopy preparation, and show that such assessments can be done via simulation frameworks, where LLMs act as medical assistants.

In this study, we selected leading LLMs to generate synthetic dialogues, where models simulated both patients and “AI Coaches” (ie, colonoscopy prep assistants). These dialogues were evaluated by both human raters and LLM-based raters, enabling us to evaluate not only the dialogue quality but also the ability of LLMs to stand in for human raters. The main benefit of synthetic data is the preservation of patient privacy and safety beyond other advantages [20]. Although LLM-generated questions may not fully capture the complexity and variability of questions that real patients may ask, they cover a broad range of topics, offering a challenge and a testing ground for models in this and other tasks [21]. The study evaluated dialogues along 2 dimensions: (1) diversity and difficulty of patient questions and (2) correctness of AI Coach responses relative to prep instructions. We aimed to better understand how close current LLMs are to serving as safe and reliable conversational assistants for real-world health care communication tasks in the context of colonoscopy procedure.

## Methods

### Dialogue Generation

Five leading LLMs with strong reasoning and instruction-following capabilities were selected for dialogue generation: OpenAI’s GPT-4.1, o3, and GPT-5.1; Meta’s Llama 3.3 70B Instruct; and Mistral’s Large-2411. GPT-4.1, o3, and GPT-5.1 are closed-weight models, whereas Mistral Large and Llama 3.3 are open-weight models. Among these, o3 and GPT-5.1 use explicit reasoning. The study was conducted from May to September 2025. GPT-5.1 was released after our data generation and human evaluation were completed. Accordingly, GPT-5.1 dialogues were generated in a separate, later phase of the study. Therefore, we report results for this newer model from automatic evaluation only.

Dialogue generation was conducted in a turn-by-turn manner, with separate prompts for patient questions and AI Coach responses (Multimedia Appendix 1). An example dialogue is provided in Table 1. For AI Coach responses, we incorporated few-shot dialogues and chain-of-thought reasoning [22], as preliminary experiments indicated that these strategies reduced errors. Few-shot examples modeled fully correct responses and illustrated how to avoid common error types. The AI Coach prompt contained full prep instructions [23] converted into raw text (Multimedia Appendix 2). Patient prompts were designed to encourage diversity and realism, so at each patient’s turn, one of several prompt types was selected at random:

- Food-related prompts generated questions about eating various foods at a specific time before the procedure or about mistakenly eating restricted foods.
- Theme-based prompts generated questions on a specific topic unrelated to foods, such as sedation, allergies, and medication.
- Follow-up prompts generated questions based on the previous turn.

**Table 1.** Dialogue example: 1. Date: 12 days before procedure. Model: GPT-4.1 Instruct.

Turns	Note
AI Coach: Hello! I’m here to help you prepare for your colonoscopy. What questions or concerns can I assist you with today?	Greeting.
Patient: Can I still eat spinach salad with vinaigrette?	Not easily answerable from instructions.
AI Coach: Yes, you can still eat spinach salad with vinaigrette. Dietary changes, such as avoiding leafy greens like spinach, start 5 days before your procedure.	Correct: reminds a patient that spinach is restricted within a 5-day window.
Patient: Got it, thanks for clarifying. What exactly is in the bowel prep drink?	Easily answerable from instructions.
AI Coach: Your bowel prep will include MiraLAX (polyethylene glycol 3350, 8.3 oz or 238 g), 4 Dulcolax tablets (5 mg each), and 64 oz of sports drink (not red or purple). These ingredients are mixed to clean your colon before the procedure.	Correct.
Patient: Will the MiraLAX irritate my ulcerative colitis?	Unanswerable from instructions.
AI Coach: Please contact your health care provider to discuss your specific medical condition and get guidance on your bowel prep.	Correct: directs the patient to a provider.

Each prompt generated 10 questions per turn, and one of them was selected at random to continue the dialogue. This is a simple approach to addressing the well-known mode collapse problem [24], where repeatedly prompting a model produces a limited set of highly similar outputs. For comparison, we also generated dialogues for each model using a baseline prompting approach, where we used a single simple prompt that produced only one question per turn. Each dialogue consisted of 3 to 7 turns (question-answer pairs), with the number of turns determined uniformly at random. A total of 250 dialogues were generated by each model using the 2 prompting approaches, with the same model acting as both the patient and the AI Coach in a dialogue. To assess potential echo-chamber effects, we performed a supplementary crossed-model analysis in which patient questions generated by Llama were answered by model o3.

In the prep instructions, dietary restrictions begin 5 days before the procedure, marking the start of the preparation period most relevant to patients. Accordingly, dialogues were assigned a time point between 1 and 21 days before the procedure, with dialogues set within the 5-day window occurring twice as frequently as those set earlier, as these tend to involve more challenging or clinically relevant questions, and patients tend to ask questions closer to the procedure. Model correctness was later stratified by short-term (1‐5 days) and longer-term (6‐21 days) windows.

### Human Evaluation

Four human raters participated in the dialogue evaluation: 1 attending physician (expert), 1 medical resident, 1 medical student (experienced raters), and 1 layperson (the first author). For each model (except GPT-5.1, as noted above), 50 dialogues were sampled at random for evaluation. A subset of 50 dialogues was annotated by all 4 raters to determine interrater agreement, with dialogues evenly distributed across models. The rest of the dialogues were split evenly between experienced raters. All analyses reported are based on expert and experienced rater annotations. Additionally, a subset of dialogues was cross-annotated by the lay rater to enable comparison between lay and experienced evaluations. To support consistency, raters were provided with detailed evaluation guidelines, which were refined following a pilot trial. All raters except the lead author were blinded to the model source of the dialogues.

Raters were instructed to determine whether each AI Coach response was correct or incorrect, with reference to prep instructions. Incorrect responses were classified into one of the following categories (Multimedia Appendix 3):

- Temporal errors: inconsistencies with the procedure timeline
- Extraneous information: inclusion of content outside the scope of prep instructions (subcategorized as factually correct vs factually incorrect)
- Reasoning errors: contradictions with prep instructions or faulty reasoning (eg, about restricted foods)
- Omissions: exclusion of essential or helpful information
- Other errors: irrelevant, disfluent, or ambiguous responses

Each error was also judged for harmfulness. An error was classified as harmful if following the advice could plausibly result in inadequate bowel preparation, violation of medication or fasting restrictions, or a canceled procedure. Errors were classified as harmless if they introduced inaccuracies that would not affect preparation quality, procedure scheduling, or patient safety (Multimedia Appendix 3). Disagreements among raters were resolved by majority vote; in cases without a consensus, the rating of the most experienced rater was used. Responses may contain multiple errors and were considered correct only if they contained none.

The correctness metric, which emphasizes strict adherence to prep instructions, was supplemented with absolute correctness, in which responses containing factually correct but extraneous information beyond the preparation instructions were merged with correct responses rather than treated as errors. Responses containing factually correct but extraneous information are harmless by definition.

Unlike AI Coach responses, patient questions were not judged for correctness because, in practice, patients may ask irrelevant or ambiguous questions, and providers are still expected to respond appropriately. Instead, 30 questions from each model were sampled at random and categorized into 1 of 3 levels: (1) easily answerable from prep instructions; (2) not easily answerable, such as those that require reasoning about the timeline or fiber content of various foods; (3) not answerable at all (eg, sedation details or anxiety management), which require deferral to a provider. This classification aimed to determine whether models were presented with questions of varying degrees of difficulty, as would be expected in a real-life scenario.

### Automatic Evaluation

We used an LLM-as-a-judge approach to evaluate a large set of dialogues, in which each AI Coach response was independently evaluated by a language model. The evaluation prompt (Multimedia Appendix 1) included the full prep instructions, few-shot examples of common errors and correct responses, and it required the model to provide an explanation whenever it judged a response as incorrect. These explanations were retained for qualitative error analysis.

Four evaluator models were tested: DeepSeek-R1, OpenAI’s GPT-5.1 and o3, and Meta’s Llama 3.3 70B. These models were selected to represent both closed-weight and open-weight models with strong reasoning performance. To determine the most reliable automated evaluator, we compared evaluator predictions with human judgments. DeepSeek-R1 demonstrated the best performance and was therefore selected as the primary automated evaluator for reporting large-scale results (Table 2). Although prior work suggests that LLM-based evaluators may exhibit self-bias [25], none of the evaluated dialogues in our study were generated by DeepSeek-R1, mitigating that risk.

**Table 2.** Performance of large language models as automated error predictors in synthetic colonoscopy preparation dialogues between simulated patients and AI Coaches (N=252)^a^.

Model	Accuracy, n (%)	Precision	Recall	*F*_1_-score
Llama 3.3 70B	199 (79.0)	0.63	0.40	0.49
GPT-5.1	207 (82.1)	0.45	0.55	0.49
o3	213 (84.5)	0.51	0.58	0.54
DeepSeek-R1	217 (86.1)	0.56	0.58	0.57

a Fifty randomly selected dialogues were cross-evaluated by expert human raters, whose annotations served as the gold standard. Model predictions of response correctness were compared against these expert ratings at the turn level. Cochran *Q* test detected no differences among evaluators.

We also implemented a simple safety filtering mechanism based on the best-performing automated evaluator. If the evaluator classified a response as incorrect, the original response was replaced with a deferral message instructing the patient to contact their health care provider for clarification. If the response was classified as correct, the original response was retained. Error and harmfulness rates were then recomputed using the modified set of responses to estimate the potential impact of this filtering mechanism. This procedure was performed offline as a postprocessing step.

Additionally, we assessed the diversity of patient questions across models using the type-token ratio (the percentage of unique unigrams and bigrams, referred to as Distinct-1 and Distinct-2, respectively) and the entropy measure. We compared question diversity generated using our multiprompt, 10-question strategy with a single-prompt, single-question approach, which served as the baseline. The most frequent tokens are listed in Multimedia Appendix 4.

### Statistical Analysis

We estimated the approximate sample size of 1228 responses per model for the automatic evaluation using a chi-square power analysis with a significance level of 0.05 and statistical power of 0.80. Pilot estimates of the observed proportions yielded a Cohen effect size of 0.094. Assuming an average of 5 responses per dialogue, 1228 responses corresponded to 245.6 dialogues per model, which was rounded to 250. A Bonferroni-adjusted significance level was applied to maintain the desired statistical power when conducting multiple comparisons. Two models (Llama 3.3 70B and GPT-5.1) produced slightly fewer responses than the planned target (1220 and 1219, respectively), representing a deviation of less than 1% from the planned sample size, which does not significantly affect the intended statistical power.

Chi-square tests and tests of proportions treat dialogue turns as independent observations. Therefore, to confirm the statistical significance of differences in turn-level accuracy between 2 models (Model A and Model B) without assuming that turns in the same dialogue were independent, we used a nonparametric permutation test at the dialogue level with a custom turn-level accuracy statistic. For each dialogue, the number of correct turns and total turns was calculated. The observed test statistic, *T*_obs_, was calculated as the difference in turn-level accuracy between Model A and Model B, which was determined by summing and dividing the correct and total turns across all dialogues for each model. We then generated an empirical null distribution by repeatedly shuffling the dialogue-level group labels (n=50,000) and recalculating the statistic for each permuted set. The 2-sided *P* value was determined as the proportion of permutations in which the absolute value of the permuted statistic was greater than or equal to |*T*_obs_|. Such pairwise permutation tests were conducted against the best-performing model with a Holm-Bonferroni–adjusted significance level of .05.

For human evaluation, we estimated the sample size required to detect significant interrater agreement using the Bloch and Kraemer formula for Krippendorff α [26], assuming a significance level of .05. However, we did not anticipate such high correctness rates observed in our data and instead calculated Gwet AC1 as the measure of interrater agreement. This statistic is more robust to skewed category distributions [27] and is reported in the *Discussion* section.

### Ethical Considerations

This study did not involve human participants; therefore, institutional board review approval was not required. All data analyzed in this study were fully synthetic and did not contain any real patient information.

## Results

### Question Difficulty and Diversity

Human evaluation of question difficulty (Table 3) showed that questions were well balanced across difficulty levels for all models, with o3 and Mistral Large generating slightly more challenging or unanswerable questions. Automatic diversity metrics, such as entropy and Distinct-1 or Distinct-2 (Table 4), indicated that the multiprompt, multiquestion generation strategy produced more diverse patient questions than the single-prompt, single-question baseline for all models. Both o3 and GPT-5.1 produced the most lexically diverse patient questions, achieving the highest Distinct-1 or Distinct-2 and entropy scores among all models. Multimedia Appendix 4 lists the most frequent tokens across models, revealing a clear qualitative pattern: stronger models such as o3, GPT-5.1, and GPT-4.1 generated more specific terms (eg, chicken, coffee, white), whereas Llama and its baseline relied more on generic ones (eg, colonoscopy, procedure). Although Mistral produced a range of specific items (eg, popcorn, wine, salad), its token frequencies were more uneven, whereas the stronger models exhibited more balanced lexical distributions.

**Table 3.** Human evaluation of patient question difficulty in colonoscopy preparation dialogues generated by different large language models (N=30 per model)^a^.

Model	Easily answerable, n	Not easily answerable, n	Unanswerable, n
Mistral Large	6	12	12
Llama 3.3 70B	10	8	12
GPT-4.1	11	10	9
o3	5	12	13

a Patient questions were generated using a multiprompt, 10 questions-per-prompt strategy. Questions were categorized by human raters according to whether they were directly answerable from the colonoscopy preparation instructions, required indirect reasoning, or were unanswerable based on the provided instructions.

**Table 4.** Automatic evaluation of question diversity in colonoscopy preparation dialogues generated by different large language models^a^.

Model	Distinct-1	Distinct-2	Entropy	N
Baseline Llama	0.03	0.10	7.22	1227
Llama 3.3 70B	0.07	0.25	8.00	1220
Baseline GPT-4.1	0.06	0.24	7.90	1264
GPT-4.1	0.11	0.39	8.59	1292
Baseline Mistral	0.09	0.28	7.91	1233
Mistral Large	0.11	0.40	8.70	1250
Baseline GPT-5.1	0.06	0.25	8.10	1282
GPT-5.1	0.16	0.47	8.93	1219
Baseline o3	0.12	0.40	9.02	1277
o3	0.16	0.51	9.14	1262

a Diversity metrics, including the proportion of unique unigrams and bigrams (Distinct-1 and Distinct-2, respectively) and lexical entropy, were computed across patient questions. Results include both a single-prompt, single-question baseline and a multiprompt, 10-questions-per-prompt generation strategy for each model.

### Overall Response Accuracy

Table 5 presents the human evaluation results for dialogue-level and turn-level accuracy relative to the colonoscopy preparation instructions. At the dialogue level, o3 achieved the highest accuracy (38/50, 76%), followed by GPT-4.1 (31/50, 62%), Llama 3.3 70B (19/50, 38%), and Mistral Large (9/50, 18%). A similar pattern was observed at the turn level, with o3 (216/231, 93.5%) significantly outperforming Llama 3.3 70B (185/237, 78.1%; *P*<.001) and Mistral Large (171/251, 68.1%; *P*<.001). The comparison with GPT-4.1 (227/250, 90.8%; *P*=.28) was not significant. When counting responses that were extraneous but still correct, absolute correctness increased only slightly for all models.

**Table 5.** Human evaluation of model performance in colonoscopy preparation dialogues generated by different large language models^a^.

Model	Dialogue-level accuracy, n/N (%)	Turn-level accuracy, n/N (%)	Absolute correctness, n/N (%)	*T*_obs_	Adjusted *P* value (vs best)
Mistral Large	9/50 (18)	171/251 (68.1)	178/251 (70.9)	25.4	<.001
Llama 3.3 70B	19/50 (38)	185/237 (78.1)	189/237 (79.7)	15.4	<.001
GPT-4.1	31/50 (62)	227/250 (90.8)	231/250 (92.4)	2.7	.28
o3	38/50 (76)	216/231 (93.5)	218/231 (94.4)	—^b^	—

a Dialogue-level and turn-level accuracy were assessed relative to publicly available colonoscopy preparation instructions from the Wexner Medical Center. Absolute correctness includes responses containing accurate but extraneous information beyond the preparation instructions. Pairwise comparisons of turn-level accuracy against the highest-performing model (o3) were conducted using dialogue-level permutation tests with Holm-Bonferroni correction.

b Not applicable.

To extend these findings to a larger dataset, we conducted an automatic evaluation using the LLM-as-a-judge approach. We used 4 LLMs as evaluators: DeepSeek-R1, OpenAI’s GPT-5.1 and o3, and Meta’s Llama 3.3 70B (Table 2). We ultimately selected DeepSeek-R1 as the evaluator, as it achieved the highest accuracy and *F*_1_-score against the human judgments. The automatic evaluation (Table 6) followed similar trends to those observed in the human evaluation. At both the dialogue level (182/250, 72.8%) and turn level (1135/1219, 93.1%), GPT-5.1 was the best-performing model, significantly exceeding o3 (158/250, 63.2% and 1145/1262, 90.7%, respectively; *P*=.04), GPT-4.1 (147/250, 58.8% and 1148/1292, 88.9%, respectively; *P*<.001), Llama 3.3 70B (104/250, 41.6% and 990/1220, 81.1%, respectively; *P*<.001), and Mistral Large (150/250, 60% and 773/1250, 61.8%, respectively; *P*<.001) at the turn level. The supplementary baseline correctness analysis (Multimedia Appendix 5) confirmed that GPT-5.1 was the best-performing model under both the single-prompt, single-question baseline and the multiprompt, multiquestion prompting approaches. In a supplementary crossed-model analysis (Table 7), o3 achieved slightly higher accuracy when answering Llama-generated patient questions (1127/1220, 92.4%) than when answering its own questions. At the dialogue level, the accuracy increased substantially by approximately 30%, suggesting that incorrect turns were concentrated within a small number of dialogues. In a supplementary stratified analysis (Multimedia Appendix 6), all models showed higher accuracy in the 6 to 21-day window at both the turn and dialogue levels. The 1 to 5-day window, sampled more frequently to reflect patient behavior in the real world, was more challenging, likely due to increased clinical complexity during the restriction period.

**Table 6.** Automatic evaluation of dialogue-level and turn-level accuracy in colonoscopy preparation dialogues generated by different large language models (LLMs)^a^.

Model	Dialogue-level accuracy, n/N (%)	Turn-level accuracy, n/N (%)	*T*_obs_	Adjusted *P* value (vs best)
Mistral Large	150/250 (60)	773/1250 (61.8)	31.3	<.001
Llama 3.3 70B	104/250 (41.6)	990/1220 (81.1)	12.0	<.001
GPT-4.1	147/250 (58.8)	1148/1292 (88.9)	4.2	.001
o3	158/250 (63.2)	1145/1262 (90.7)	2.4	.04
GPT-5.1	182/250 (72.8)	1135/1219 (93.1)	—^b^	—

a Accuracy was determined using an LLM-as-judge approach, with DeepSeek-R1 serving as the automated evaluator. Pairwise comparisons of turn-level accuracy against the highest-performing model (GPT-5.1) were conducted using dialogue-level permutation tests with Holm-Bonferroni correction.

b Not applicable.

**Table 7.** Supplementary automatic evaluation of dialogue-level and turn-level accuracy in colonoscopy preparation dialogues for Llama 3.3 70B and o3 under original and crossed dialogue-generation conditions^a^.

Model	Dialogue-level accuracy, n/N (%)	Turn-level accuracy, n/N (%)
o3	158/250 (63.2)	1145/1262 (90.7)
Llama+o3	232/250 (92.8)	1127/1220 (92.4)

a Accuracy was determined using an LLM-as-judge approach, with DeepSeek-R1 serving as the automated evaluator. Dialogues generated entirely by o3 were compared with a crossed-model condition in which patient questions generated by Llama 3.3 70B were answered by o3.

### Error Analysis

Error breakdowns are reported in Table 8. The most frequent error type across models was omission, ranging from 1.7% (4/231) for o3 to 11.6% (29/251) for Mistral Large. Models o3 and GPT-4.1 consistently had the lowest error rates across all categories. The error rates of model o3 remained uniformly below 2% across all error types. Table 9 shows that both the number and proportion of harmful errors varied notably by model. Mistral Large produced the highest number of total errors but surprisingly a lower proportion of harmful errors (37/114, 32.5%). In contrast, model o3 had the fewest total errors but the highest proportion that were harmful (11/17, 64.7%).

**Table 8.** Distribution of error types across models in colonoscopy preparation dialogues generated by different large language models based on human evaluation^a^.

Model	Temporal, n/N (%)	Extraneous correct, n/N (%)	Extraneous incorrect, n/N (%)	Reasoning, n/N (%)	Omission, n/N (%)	Other, n/N (%)
Mistral Large	26/251 (10.4)	8/251 (3.2)	7/251 (2.8)	27/251 (10.8)	29/251 (11.6)	3/251 (1.2)
Llama 3.3 70B	10/237 (4.2)	4/237 (1.7)	9/237 (3.7)	7/237 (3.0)	23/237 (9.7)	3/237 (1.3)
GPT-4.1	7/250 (2.8)	4/250 (1.6)	3/250 (1.2)	1/250 (0.4)	12/250 (4.8)	1/250 (0.4)
o3	1/231 (0.4)	3/231 (1.3)	4/231 (1.7)	4/231 (1.7)	4/231 (1.7)	1/231 (0.4)

a Error categories were defined according to predefined annotation guidelines. Fifty dialogues per model were evaluated. Rates are calculated at the turn level, and n denotes the number of turns containing at least 1 error of the specified type.

**Table 9.** Proportion of harmless vs harmful errors across models in colonoscopy preparation dialogues generated by different large language models, based on human evaluation^a^.

Model	Harmless, n/N (%)	Harmful, n/N (%)
Mistral Large	77/114 (67.5)	37/114 (32.5)
Llama 3.3 70B	28/58 (48.3)	30/58 (51.7)
GPT-4.1	17/28 (60.7)	11/28 (39.3)
o3	6/17 (35.3)	11/17 (64.7)

a Harmfulness was defined as an error with the potential to cause clinically significant consequences, including procedure cancellation or adverse health outcomes.

## Discussion

### Diversity of Patient Questions

Prompt design had a clear effect on the diversity of patient queries. A simple baseline prompt produced repetitive questions, whereas our multiprompt strategy, where each turn generated 10 candidate questions from food-related, thematic, or follow-up prompts, led to greater lexical and topical variety, helping mitigate the mode collapse problem. Automatic metrics (Distinct-*n* and entropy) confirmed this increase in diversity. Human evaluation further showed that our approach yielded a balanced distribution of questions: some were easily answerable from the preparation instructions, others required indirect reasoning (eg, about timing or food composition), and some were not answerable at all (eg, anxiety or sedation concerns). This suggests that our generation setup exposed models to a realistic range of question types and difficulty, mirroring those encountered in actual patient-provider interactions. Consequently, the resulting dialogues provide a more robust basis for evaluating models’ performance in this task.

### Correctness of AI Coach Responses

This task is relatively straightforward: responses are evaluated against a fixed set of colonoscopy preparation instructions. However, the best-performing models (GPT-4.1, o3, and GPT-5.1) approach but do not reach 100% accuracy. Smaller open-source models (Llama and Mistral) are not a viable alternative due to a substantially higher number of errors. While a fully crossed design could be considered in future work, the preliminary analysis of the Llama and o3 pairing indicates that using the same-model pairing did not artificially boost performance.

Error patterns revealed important distinctions across models. Temporal errors were much less frequent in the stronger models; however, instruction-following errors (omission, extraneous information, and faulty reasoning) remained prevalent even when temporal accuracy was high, suggesting that models could benefit from further fine-tuning for instruction adherence. Additionally, o3 produced a large proportion of harmful errors despite high overall accuracy, showing that accuracy and safety are not equivalent. In fact, the existence of harmful errors demonstrates the potential risks of deploying even highly accurate models in patient-facing contexts without additional safeguards. This concern may be especially relevant in scenarios where patients already have a solid understanding of preparation instructions, since models could inadvertently misdirect otherwise well-prepared patients. For less-prepared patients, however, model assistance could still be mildly beneficial, although not enough on its own to ensure a successful prep. Patients may need reminders [3] to complete preparation steps, which would in turn require a more complex interactive system capable of supporting these mechanisms.

Common harmful errors involved omissions of critical safety or procedural information. Examples include failing to mention the need for a designated driver at check-in, omitting the 2-hour restriction on liquids (with the exception of small sips of water for medication), or failing to warn about future dietary restrictions (eg, avoiding red or purple liquids and dairy products on the day before the procedure). Other errors concerned medication guidance, such as not advising patients to consult their provider about medication changes or neglecting to state specific rules for oral diabetes medications. In several cases, models failed to instruct patients to contact their provider after mistakenly consuming a prohibited food item. There were also some patient questions phrased in terms of weekdays (eg, “this Sunday”) rather than in terms of the number of days left before the procedure (eg, “3 days before”). Such questions create ambiguity that a robust system should detect, but even stronger models were not able to do so.

Model behavior also differed in style. Mistral’s responses were often extremely brief, offering no explanation derived from the instructions and sometimes consisting of single-word replies. Mistral also refused to answer many dietary questions when dialogues were set many days before the procedure and conflated patient and provider roles more often than others. Llama tended to be verbose and occasionally unnatural in phrasing, whereas GPT and o3 produced more natural responses, which were lengthy only when warranted.

### Evaluation

Both automatic and human evaluation were essential and complementary. Human evaluation provided insights into error types and their potential harmfulness, while automatic evaluation provided a scalable way to evaluate large numbers of dialogues beyond what would be feasible with expert raters. Interrater agreement for correctness judgments among the human raters was substantial (expert and experienced raters: AC1=0.74, 95% CI 0.68-0.80, percent agreement=0.82; all raters: AC1=0.68, 95% CI 0.62-0.75, percent agreement=0.79), consistent with our expectation that evaluating factual correctness in this task is easier than clinical diagnosis.

Interestingly, we found systematic differences between DeepSeek-R1 (R1) and the expert raters. R1’s strict adherence to prep instructions led it to identify certain erroneous responses that the experts judged acceptable, suggesting that automatic evaluators may in fact be more reliable at guideline and prep fidelity. Some of these cases involved minor issues, such as responses that were incomplete or lacked explanation. In one case, the response model misclassified Tylenol as a nonsteroidal anti-inflammatory drug (NSAID), which constitutes a factual error but not a harmful one, as experts agreed the real safety concern lies with anticoagulants such as Warfarin. In another case, the response model failed to specify that instant Ramen should be made from low-fiber noodles. Although technically correct, this omission was considered inconsequential because most instant Ramen products already meet that criterion. R1 was able to successfully identify these erroneous cases, but it also penalized a few factually correct responses, reflecting an overly rigid standard of adherence.

These findings do not undermine the earlier results from human evaluation. Importantly, evaluating a large set of responses offline in a spreadsheet differs from interacting with real patients, where pragmatic communication takes precedence. The lay rater showed similar tendencies to R1, prioritizing strict adherence while making only a single factual error (incorrectly believing that gummy bears were allowed on the day before the procedure). This further suggests that factual evaluation of dialogues in reference to prep instructions is a relatively easy task for humans to learn, yet even the strongest models are not perfect at it.

Regarding false negatives, R1 missed a few temporal and reasoning errors, but no clear pattern was observed among them. In a safety filtering context, such false negatives are more concerning than false positives because they allow incorrect responses to remain unflagged. However, these cases were relatively infrequent and do not change the comparative findings across models.

### Safety Filter

Applying an LLM-as-a-judge filter (Table 10) to the human-annotated subset of dialogues reduced both the overall error rate and the harmfulness rate for all models. The filter consisted of replacing all AI Coach responses that the LLM judge classified as incorrect with a deferral to a provider, then recomputing turn-level error and harmfulness rate on the responses. The relative improvement was greatest for Mistral Large (error rate reduced from 80/251, 31.9% to 27/251, 10.8%) and Llama 3.3 70B (52/237, 21.9% to 29/237, 12.2%). Model o3 remained the most accurate before and after filtering (15/231, 6.5% to 12/231, 5.2%). Similarly for harmfulness, the filter helped weaker models like Llama and Mistral, but offered little or no benefit for stronger models such as GPT-4.1 or o3, where it removed only one harmful error per model. These improvements should be interpreted with caution, since we do not penalize false provider deferrals in our framework. If such a filter were deployed in patient interactions, it would likely be counterproductive: if patients are redirected to contact a provider for questions that could have been answered from the prep instructions, the usefulness of the AI Coach as an assistant for colonoscopy prep is undermined. Most importantly, the filter failed to eliminate a sufficient number of harmful errors, making it impractical as a safety mechanism.

**Table 10.** Error and harmfulness rates in the human-annotated subset of colonoscopy preparation dialogues before and after applying a large language model (LLM)-as-a-judge filter^a^.

Filtering	Before		After	
Model	Error rate, n/N (%)	Harmfulness, n/N (%)	Error rate, n/N (%)	Harmfulness, n/N (%)
Mistral Large	80/251 (31.9)	33/251 (13.1)	27/251 (10.8)	11/251 (4.4)
Llama 3.3 70B	52/237 (21.9)	30/237 (12.7)	29/237 (12.2)	18/237 (7.6)
GPT-4.1	23/250 (9.2)	9/250 (3.6)	15/250 (6)	8/250 (3.2)
o3	15/231 (6.5)	10/231 (4.3)	12/231 (5.2)	9//231 (3.9)

a The filter replaced AI Coach responses classified as incorrect by the LLM judge with a deferral advising the patient to contact their provider. Rates are calculated at the turn level; N denotes the number of turns containing at least one error and at least one harmful error, respectively.

### Limitations

This study has several limitations. First and foremost, the use of synthetic dialogues may limit external validity. The distribution of synthetic patient questions may not fully capture the range or underlying motivations behind real-world patient inquiries. The factors driving patient unpreparedness are not fully understood and can stem from causes beyond informational gaps, such as anxiety or noncompliance, that are difficult to model through prompt-based generation. Additionally, real patient questions can prove to be more challenging, as they can be emotionally loaded, unclear, or fragmented and may require follow-up clarification, although expert raters qualitatively found the synthetic questions to be otherwise plausible. Furthermore, the prompts explicitly encouraged creativity, which may have led to overrepresentation of atypical or edge-case questions that do not fully reflect the distribution of real patient inquiries. Consequently, models might perform differently when confronted with real patient queries along with potential medical comorbidities, and the correctness results reported here may not directly translate to real clinical deployment settings. Future evaluation on real patient-provider communication data will be necessary to determine the true clinical generalizability of our findings.

Second, although model responses were evaluated for factual accuracy relative to the preparation instructions, other aspects of communication quality, such as empathy, were not assessed. Additionally, while a crossed-model analysis was performed to partially assess potential echo-chamber effects, this was limited to a subset of model pairings and does not exclude such effects for weaker models. In the future, all models should be evaluated against a standardized, independent set of questions. Although automated evaluation enabled large-scale analysis, results should be interpreted with caution, even as model rankings were preserved relative to human evaluation. The automated evaluator showed moderate agreement with human judgments (*F*_1_-score=0.57), with corresponding false positive and false negative rates that introduce noise into correctness estimates. However, such noise has always been inherent to automated evaluation [28-30] and is mitigated by the large sample size, which improves the stability of relative comparisons despite reduced precision at the individual-turn level.

Third, this study did not explore the relationship between question difficulty and response correctness or harmfulness. Fourth, diversity measures like type-token ratio and entropy quantify surface-level lexical variety but do not necessarily reflect semantic or pragmatic diversity. In addition, patient personas were defined generically and did not explicitly incorporate comorbidities or medication use, limiting assessment of model performance in more clinically complex scenarios. We did not have access to a corpus of messages or phone call transcripts between patients and providers as these are privacy-protected; consequently, we were not able to quantitatively compare the synthetic conversations with real ones. Finally, because the colonoscopy preparation instructions used in prompts are publicly available, some models may have encountered them during pretraining. As a result, part of the observed performance could be inflated and reflect memorization rather than strictly reasoning from the prompts.

### Conclusions

Taken together, our results demonstrate that LLMs approach but do not yet achieve adequate performance in this task, given the number of harmful errors. Human and automatic evaluations together provide a nuanced understanding of model behavior, balancing interpretability and scalability. Prompt-based automatic filtering improves performance only for open models and does not fully prevent harmful errors, suggesting that the practical benefits of such straightforward methods remain limited. Future work should explore a variety of approaches for reducing the number of harmful errors, such as improving evaluator models through self-training [31] or fine-tuning generator models to improve response quality [32-35]. Another promising direction is calibrating an evaluator model’s confidence: overly cautious models risk unnecessary deferrals, while overconfident ones can allow harmful misinformation to pass through. Future research should aim to develop adaptive systems capable of calibrating their confidence based on the context and risk level of a patient’s question. Future safety measures could also investigate rule-based medical constraint checking [11]. Finally, testing on real patient queries will be necessary to validate model evaluation and help align models more closely with real patient expectations.

## Supplementary material

10.2196/88581 Multimedia Appendix 1 Prompt examples.

10.2196/88581 Multimedia Appendix 2 Prep instructions.

10.2196/88581 Multimedia Appendix 3 Error examples.

10.2196/88581 Multimedia Appendix 4 Most frequent tokens.

10.2196/88581 Multimedia Appendix 5 Baseline correctness.

10.2196/88581 Multimedia Appendix 6 Correctness by temporal window.

## Abbreviations

CRAFT-MD: Conversational Reasoning Assessment Framework for Testing in Medicine
EHR: electronic health records
FAQ: frequently asked question
GRADE: Grading of Recommendations Assessment, Development, and Evaluation
LLM: large language model
NSAID: nonsteroidal anti-inflammatory drug
