# Supplementary material for: Correctness, Harmfulness, and Diversity of Large Language Models for Colonoscopy Preparation Assistance: Comparative Evaluation Study
Source: JMIR AI. 2026 Aug 4;5:e88581. doi: 10.2196/88581 (PMC13436793; doi:10.2196/88581)
Supplement: Supplementary file 2 [file ai-v5-e88581-s002.docx]

# **Multimedia Appendix 2: Prep Instructions**

This is a Multimedia Appendix to a full manuscript published in the J Med Internet Res. For full copyright and citation information see http://dx.doi.org/10.2196/jmir.88581.

The Wexner Medical Center at The Ohio State University has published several colonoscopy preparation instruction sets. The version used in this study corresponds to the one-day bowel preparation with MiraLAX and Dulcolax [1]. It was converted into plain text for use in the prompts in June 2025, at the time of the study, as follows:

[1] You will need to do a bowel prep with MiraLAX and Dulcolax before your colonoscopy. As part of the prep, you may need to make changes to your medicines many days before your procedure. These include blood thinners, medicines for diabetes, weight loss, heart conditions, and others. If you do not make the needed medicine changes, your procedure may need to be rescheduled. You will need to change your diet starting 5 days before the procedure and start the bowel prep at 3 p.m. the day before the procedure. You need to have an adult come with you to your procedure. If there are major changes to your health, please contact the health care provider who is doing your procedure. Your procedure may need to be changed for your safety. If you have other questions or concerns about your prep for your procedure, please also contact the health care provider who is doing your procedure.

[2] You need to clean out your colon before the procedure so the doctor can see your colon. This means you will need to purchase in advance a large bottle of laxative polyethylene glycol 3350 such as Miralax (8.3 ounces or 238 grams), 4 tablets of Dulcolax laxative (5 mg each) and a total of 64 ounces of sports drinks, like Gatorade. The drinks cannot be red or purple in hue. Select a sugar-free option if you have diabetes. You will also need to adjust your diet before the procedure. Starting 5 days before your procedure, you should not eat: corn, nuts, leafy greens, popcorn, fruit with skin or seeds, whole grain or high fiber foods.

[3] You may need to make changes to your medicines many days before your procedure. Tell your doctor all the medicines you take, including vitamins, supplements, and herbal remedies. Your doctor will tell you if you should stop taking any of them before this procedure and how soon to do it. If you take aspirin or NSAIDs, such as ibuprofen (Advil, Motrin, and Nuprin), naproxen (Aleve), or celecoxib (Celebrex) for pain, you do not need to stop taking these medicines before this procedure. You may not take vitamins with iron, iron tablets, or fiber supplements. If you take oral medicines for diabetes, you should not take oral diabetes medicines on the day of your procedure. If you take injection medicines for diabetes, such as insulin, ask the doctor who ordered the medicines how to adjust your medicines for this procedure.

[4] If you take a blood thinner (anticoagulant or antiplatelet) medicine, ask the doctor who ordered this medicine if it is safe for you to stop taking it before this procedure. The doctor may or may not tell you to stop taking it a certain number of days before your procedure. Make sure that you understand exactly what your doctor wants you to do. If you have had a stent, especially a stent in your heart or brain, you should not stop taking your blood thinner/stent medicine until you are instructed by the doctor who placed the stent. If your doctor has told you not to stop taking these medicines before this procedure, please contact the health care provider who is doing your procedure.

[5] You will drink a medicine mixture to clear your bowels of all solid matter. You will need to go to the bathroom often, and your stool will get very watery. The prep may cause you to have cramps or feel bloated. The prep medicine may not taste good. You need to take all of it so your bowels are clear. Your bowels are clear when you are passing pale yellow liquid without any stool. If you vomit up your prep medicine, have not had any bowel movements, or your bowels are not clear after this prep, please contact the health care provider who is doing your procedure. The procedure may need to be rescheduled. If you have a history of heart failure, kidney disease, cirrhosis of the liver, electrolyte problems, or chronic constipation, you may need a different bowel prep medicine. Please contact the nurse for instructions.

[6] The day before your procedure you will start a clear liquid diet. You may not eat any solid food or eat or drink any milk products until after your procedure is done. You may drink only clear or light colored liquids. You should not have anything with red or purple dye. It is okay to have: water, flavored water, or ice chips; coffee or tea (no milk or cream); ginger ale or lemon-lime soda; fruit juices you can see through, such as apple or white grape; Gatorade, other sports drinks, or drink mixes like Kool-Aid; popsicles that aren’t red or purple (no fruit or cream); Jell-O or other gelatin, without fruit (no red or purple); clear broth or bouillon. You may not have: alcoholic drinks; milk; smoothies; milkshakes; cream; yogurt; orange juice; grapefruit juice; tomato juice; soup (other than clear broth); red or purple drinks. You should also not smoke or vape the day before the procedure and on the day of the procedure.

[7] At 3 p.m. on the day before your procedure, take 4 Dulcolax tablets. Pour all of the MiraLAX and the two bottles of sports drink into a larger container and shake well. Put it into the refrigerator to keep cool. At 6 p.m. on the day before your procedure, drink one (8-ounce) glass of the mixture every 10 to 15 minutes until you finish half of it. It may help to use a straw. Put the rest back in the refrigerator. 6 hours before your procedure, drink the rest of the mixture. You may need to set your alarm to get up to finish your prep medicine. Drink two (8-ounce) glasses of clear liquids like water after you finish the mixture. You can drink clear liquids up to 4 hours before your procedure.

[8] You can take a small sip of water to take any allowed medicines within 2 hours of your procedure. Your procedure will be delayed or canceled if you drink anything other than a sip of water with medicines. This is important to make sure it is safe to give you sedation or anesthesia. If you have alcohol, marijuana or illegal drugs in your system, your procedure may be rescheduled for your safety.

[9] Arrive by the arrival time on your letter, so the staff can get you ready. Expect to be here 2 to 4 hours for your procedure and recovery time. Please leave all valuables and jewelry, including piercings, at home. Patients who can become pregnant should come prepared to give a urine sample in case a pregnancy procedure is needed. Bring these with you to your procedure: list of all medicines, including prescription and over the counter medicines, and any vitamin or herbal products you are taking; list of your allergies; list of medical conditions and previous surgeries; copy of your advanced directive, such as a living will or power of attorney; photo identification, insurance card, and co-payment, if needed.

[10] You will be given medicine to help you relax during the procedure. For your safety, you will need a responsible adult to drive you home after the procedure. Your driver needs to check in for the procedure with you. If your driver doesn’t check in with you, we will need to speak to your driver before we can start your procedure. Your procedure will be delayed or canceled if we cannot speak to your driver. If your driver leaves the facility during the procedure, they need to give the staff a phone number where they can be reached. Your driver needs to be within 30 minutes of the procedure location. If you take a cab, bus, or medical transportation, an adult other than the driver needs to ride with you for your safety. You should have an adult with you to help you at home after the procedure for at least 6 hours. You should not drive, operate machinery, drink alcohol, or make any legal decisions until the day after your procedure.

# **References**

[1] OSU WMC. 1 Day Bowel Prep With MiraLAX and Dulcolax. [https://www.healthwise.net/osumychart/ Content/StdDocument.aspx?DOCHWID=custom.hs0146](https://www.healthwise.net/osumychart/Content/StdDocument.aspx?DOCHWID=custom.hs0146) 2025. Accessed: 2025-06-06.
