# Supplementary material for: Correctness, Harmfulness, and Diversity of Large Language Models for Colonoscopy Preparation Assistance: Comparative Evaluation Study
Source: JMIR AI. 2026 Aug 4;5:e88581. doi: 10.2196/88581 (PMC13436793; doi:10.2196/88581)
Supplement: Supplementary file 4 [file ai-v5-e88581-s004.docx]

# **Multimedia Appendix 4: Most Frequent Tokens**

This is a Multimedia Appendix to a full manuscript published in the J Med Internet Res. For full copyright and citation information see http://dx.doi.org/10.2196/jmir.88581.

In processing the patient questions, we filtered out articles (e.g., the, a, an), prepositions (e.g., about, on, in, except those semantically relevant to the task, such as before), pronouns (e.g., she, I, you), auxiliary and modal verbs (e.g., would, can, should), interrogatives (e.g., who, what), common interjections (e.g., thanks, okay), and other frequent non-informative words.

**Table S1:** Top 25 most frequent tokens and their counts of synthetic patient questions generated by large language models in colonoscopy preparation dialogues between simulated patients and AI Coaches. Results include both a single-prompt, single-question baseline for Llama and a multi-prompt, 10-questions-per-prompt generation strategy for each model.

| Baseline Llama | Llama 3.3 70B | Mistral Large | GPT-4.1 | o3 | GPT-5.1 |
| --- | --- | --- | --- | --- | --- |
| procedure: 859 | procedure: 499 | days: 304 | colonoscopy: 165 | feeling: 128 | nervous: 129 |
| after: 690 | days: 287 | before: 185 | procedure: 141 | prep: 100 | prep: 100 |
| take: 319 | before: 248 | procedure: 183 | prep: 126 | nervous: 87 | clear: 100 |
| colonoscopy: 276 | after: 179 | see: 153 | eat: 118 | clear: 61 | drink: 79 |
| home: 234 | ago: 178 | eat: 141 | nervous: 117 | plain: 59 | coffee: 67 |
| long: 225 | eat: 178 | glass: 118 | feeling: 116 | eat: 55 | chicken: 60 |
| drive: 221 | eating: 168 | bowl: 111 | before: 114 | procedure: 54 | plain: 59 |
| feeling: 213 | whole: 161 | salad: 107 | feel: 88 | p.m: 49 | before: 58 |
| completely: 203 | process: 152 | dinner: 102 | salad: 83 | after: 49 | white: 57 |
| sedation: 196 | colonoscopy: 152 | prep: 97 | breakfast: 74 | before: 49 | seeds: 57 |
| specific: 193 | anxious: 150 | chicken: 94 | relief: 73 | morning: 48 | ate: 56 |
| follow: 187 | prep: 144 | coffee: 94 | avoid: 73 | allowed: 47 | day: 56 |
| discomfort: 181 | feeling: 143 | enjoy: 88 | tomorrow: 72 | helps: 46 | red: 55 |
| whole: 181 | take: 134 | ago: 85 | make: 69 | sip: 45 | black: 54 |
| aware: 170 | able: 125 | good: 83 | clear: 69 | call: 45 | allowed: 50 |
| anxious: 161 | getting: 124 | patient: 83 | after: 66 | tomorrow: 44 | procedure: 49 |
| process: 148 | drink: 119 | after: 82 | problem: 63 | water: 44 | sports: 48 |
| complications: 130 | foods: 107 | popcorn: 81 | days: 62 | drink: 44 | anxious: 47 |
| normal: 126 | relief: 104 | drink: 78 | know: 60 | days: 43 | broth: 47 |
| pain: 123 | like: 100 | wine: 77 | chicken: 58 | soup: 42 | pm: 46 |
| experience: 122 | issues: 100 | breakfast: 77 | start: 57 | great: 42 | helps: 44 |
| asleep: 120 | avoid: 98 | day: 75 | drink: 56 | coffee: 42 | days: 43 |
| ensure: 115 | specific: 97 | morning: 73 | get: 56 | acceptable: 41 | cream: 43 |
| kind: 114 | affect: 95 | soup: 72 | right: 56 | anxious: 40 | tomorrow: 42 |
| restrictions: 113 | worried: 95 | avoid: 70 | oatmeal: 55 | day: 40 | juice: 41 |
