# Supplementary material for: Correctness, Harmfulness, and Diversity of Large Language Models for Colonoscopy Preparation Assistance: Comparative Evaluation Study
Source: JMIR AI. 2026 Aug 4;5:e88581. doi: 10.2196/88581 (PMC13436793; doi:10.2196/88581)
Supplement: Supplementary file 5 [file ai-v5-e88581-s005.docx]

# **Multimedia Appendix 5: Baseline Correctness**

This is a Multimedia Appendix to a full manuscript published in the J Med Internet Res. For full copyright and citation information see http://dx.doi.org/10.2196/jmir.88581.

Across models, the multi-prompt, multi-question generation strategy was associated with higher turn-level accuracy (except for Mistral and Llama) but lower dialogue-level accuracy compared with the single-prompt, single-question baseline. A likely contributing factor is that the baseline approach produces highly repetitive questions, which could artificially inflate or deflate accuracy depending on whether repeated questions are consistently easy or difficult. GPT-5.1 remained to be the best-performing model at both dialogue- and turn-level accuracy under both prompting approaches.

**Table S1:** Automatic evaluation of dialogue- and turn-level accuracy in colonoscopy preparation dialogues generated by different large language models. Results include both a single-prompt, single-question baseline and a multi-prompt, 10-questions-per-prompt generation strategy. Accuracy was determined using an LLM-as-judge approach, with DeepSeek-R1 serving as the automated evaluator.

| Model | Dialogue-level Accuracy, n/N (%) | Turn-level Accuracy, n/N (%) |
| --- | --- | --- |
| Baseline Mistral | 159/250 (63.6) | 779/1233 (63.2) |
| Mistral Large | 150/250 (60) | 773/1250 (61.8) |
| Baseline Llama | 226/250 (90.4) | 1087/1227 (88.6) |
| Llama 3.3 70B | 104/250 (41.6) | 990/1220 (81.1) |
| Baseline GPT-4.1 | 208/250 (83.2) | 1087/1264 (86) |
| GPT-4.1 | 147/250 (58.8) | 1148/1292 (88.9) |
| Baseline o3 | 221/250 (88.4) | 1138/1277 (89.1) |
| o3 | 158/250 (63.2) | 1145/1262 (90.7) |
| Baseline GPT-5.1 | **227/250 (90.8)** | 1151/1282 (89.8) |
| GPT-5.1 | 182/250 (72.8) | **1135/1219 (93.1)** |
