# Supplementary material for: Correctness, Harmfulness, and Diversity of Large Language Models for Colonoscopy Preparation Assistance: Comparative Evaluation Study
Source: JMIR AI. 2026 Aug 4;5:e88581. doi: 10.2196/88581 (PMC13436793; doi:10.2196/88581)
Supplement: Supplementary file 6 [file ai-v5-e88581-s006.docx]

# **Multimedia Appendix 6: Correctness by Temporal Window**

This is a Multimedia Appendix to a full manuscript published in the J Med Internet Res. For full copyright and citation information see http://dx.doi.org/10.2196/jmir.88581.

**Figure S1:** Dialogue-level accuracy of colonoscopy preparation dialogues generated by large language models, automatically evaluated by an LLM judge. Each dialogue was assigned a simulated time point between 1 and 21 days before the procedure, with dialogues set within the 5-day window occurring twice as frequently as those set earlier, as these tend to involve more challenging or clinically relevant questions, and patients tend to ask questions closer to the procedure. Accuracy is reported for two temporal windows: 1–5 and 6–21 days before the procedure.

**Figure S2:** Turn-level accuracy of colonoscopy preparation dialogues generated by large language models, automatically evaluated by an LLM judge. Each dialogue was assigned a simulated time point between 1 and 21 days before the procedure, with dialogues set within the 5-day window occurring twice as frequently as those set earlier, as these tend to involve more challenging or clinically relevant questions, and patients tend to ask questions closer to the procedure. Accuracy is reported separately for two clinically relevant temporal windows: 1–5 and 6–21 days before the procedure.
